# Switchable dual-mode nanolaser: mastering emission and invisibility through phase transition materials

**DOI:** 10.1515/nanoph-2023-0249

**Published:** 2023-09-01

**Authors:** Sergey Lepeshov, Andrey Vyshnevyy, Alex Krasnok

**Affiliations:** Department of Electrical and Photonics Engineering, DTU Electro, Technical University of Denmark , DK-2800 Kgs. Lyngby, Denmark; Emerging Technologies Research Center, XPANCEO, Dubai Investment Park 1, Dubai, United Arab Emirates; Department of Electrical and Computer Engineering, Florida International University, Miami, FL 33174, USA; Knight Foundation School of Computing and Information Sciences, Florida International University, Miami, FL 33199, USA

**Keywords:** nanolaser, invisibility, phase transition materials

## Abstract

The principle of detailed balance states that objects efficiently emitting radiation at a specific wavelength also efficiently absorb radiation at the same wavelength. This principle presents challenges for the design and performance of photonic devices, including solar cells, nanoantennas, and lasers. A design that successfully integrates the properties of an efficient emitter in one state and invisibility in another state is essential for various applications. In this work, we propose a novel nanolaser design based on a semiconductor nanoparticle with gain enveloped by a phase transition material that enables switching between lasing and cloaking (nonscattering) states at the same operating frequency without modifying the pumping conditions. We thoroughly investigate the operational characteristics of the nanolaser to ensure optimal performance. Our nanolaser design can function with both optical and electric pumping and exhibits the features of a thresholdless laser due to its high beta-factor and strong Purcell enhancement in the tightly confined Mie resonance mode. Additionally, we develop a reconfigurable metasurface comprising lasing-cloaking metaatoms capable of transitioning from lasing to a nonscattering state in a fully reversible manner.

## 1 Introduction

The principle of detailed balance states that efficient light emitters must also strongly interact with light through enhanced absorption and scattering [1–6]. This principle is fundamental for microwave, terahertz (THz), and optical emitters, as it requires equal radiation and reception efficiencies [7–9]. It also leads to Kirchhoff’s law, connecting spectral emissivity and absorptivity for systems in thermal equilibrium [10]. This principle enables dual devices, such as receiving-transmitting antennas and radar systems [7], emitting–absorbing nanoantennas [11], and optically pumped laser LEDs [12]. However, these interactions impose fundamental constraints on optical device performance across the spectrum. For example, solar cells with high absorption often face reduced conversion efficiency due to increased thermal radiation, while low-emissivity cells compromise solar energy absorption [13–15]. An ideal solar cell would also be a perfect LED, but the detailed balance prevents this [5]. Overcoming these limitations typically involves nonreciprocal materials based on using strong magnetic field biases, optical nonlinearity, or time-variation methods [2]. Alternatively, distinguishing between light emission and absorption/scattering effects in the frequency or time domain can be employed in a tunable or reconfigurable system.

Modern trends in miniaturization require nanophotonic devices to work in both light-emitting and light-absorbing regimes while becoming invisible to avoid interference with other elements. The shift to nanoscale optical devices has led to the development of nanolasers, a new class of coherent emitters. The concept of surface plasmon amplification by stimulated emission of radiation (SPASER), introduced by David Bergman and Mark Stockman in 2003, marked the beginning of spasers and nanolasers [16]. Numerous designs have since emerged [17], with noble metal nanoparticle-encased active media being particularly promising for optimal overlap between material gain and lasing mode [18–22]. More recently, semiconductor-based nanolasers have become low-threshold, efficient alternatives to plasmonic spasers [23, 24], with perovskites and quantum dot (QD) inclusions attracting attention for their high material gain and optical activity [25–28]. Nanolasers have been successful in applications such as sensing, biological probing, super-resolution imaging, vortex beam generation, on-chip integrated optical interconnects, and all-optical data processing [19].

However, nanolasers show enhanced scattering, especially at the lasing threshold [28], which imposes limitations on their use in optical interconnections and sensing. Differentiating light emission from absorption and scattering phenomena in nanolasers is challenging due to the inability of existing nanolaser designs to shift between various scattering states. They are restricted to transitioning between narrowband laser emissions and broadband resonant or nonresonant scattering by modifying the pump intensity. Recently proposed anapolar lasers have gained attention due to their potential to lower the lasing threshold intensity in configurations that utilize optical pumping [29, 30]. Transitioning this nanolaser to another scattering mode, like one with suppressed scattering from an anapole, necessitates turning off optical pumping, fundamentally altering the system. All this raises the question: *can a nanolaser be cloaked without modifying its pumping conditions?*

**Figure 1:**
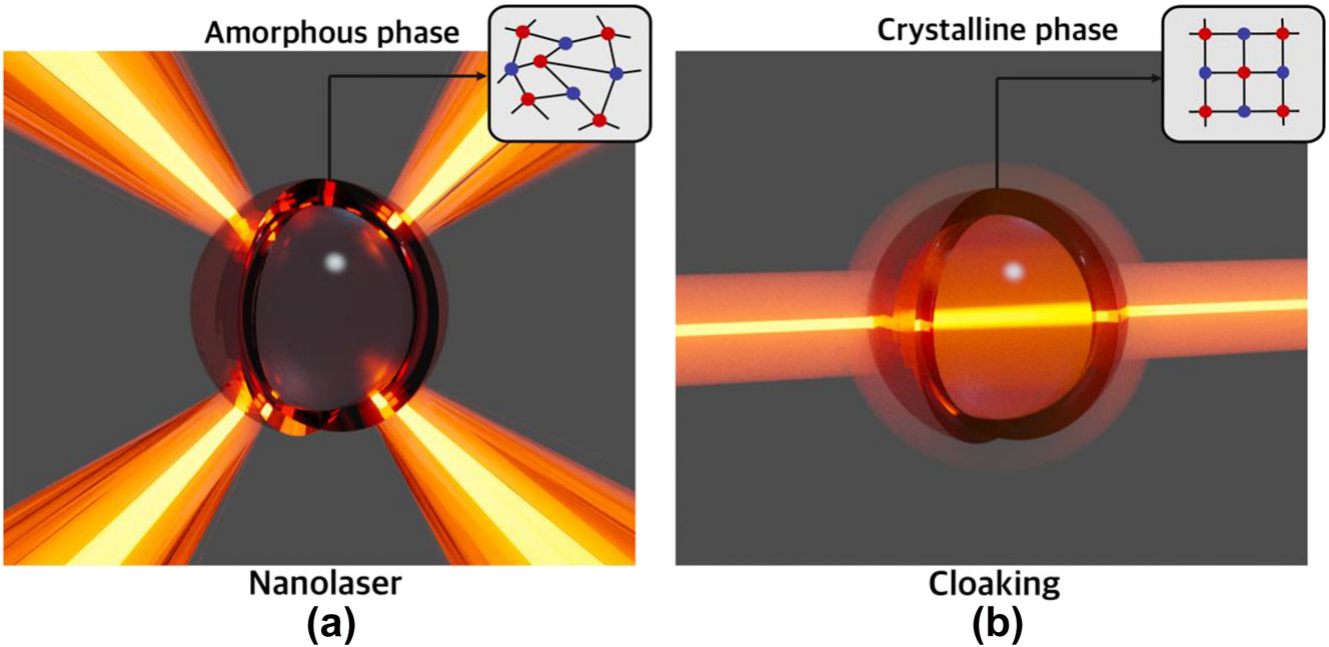
Cloaking a nanolaser. Illustration of the core–shell nanostructure supporting reversible switch between (a) nanolaser regime and (b) cloaking (anapole) regime at the same frequency.

In this work, we present a novel method that enables a nanolaser to switch between emission and cloaking (nonscattering) modes at the same operating frequency. Our design features a core–shell nanostructure composed of a semiconductor nanoparticle (NP) enveloped by a phase change material (PCM) [31–33]. The PCM shell enables the refractive index change via the transition between amorphous and crystalline states. This unique nanostructure allows reversible switching between two optical states: coherent light emission [Figure 1(a)] and cloaking [Figure 1(b)]. The cloaking phase is linked to the excitation of an anapole state [34–36]. We investigate the dynamics of the proposed tunable nanolaser in the frequency domain and input-output characteristics in the stationary state. Additionally, by utilizing this single lasing-cloaking metaatom, we develop a reconfigurable metasurface capable of reversibly transitioning from laser radiation to a nonscattering state. We demonstrate that this intelligently designed nanolaser can indeed be cloaked and subsequently restored to the laser state without altering its pumping conditions.

## 2 Results and discussion

We consider a spherical core–shell nanostructure made of a semiconductor core with material gain and an antimony trisulfide (Sb_2_S_3_) shell. Sb_2_S_3_ is a prospective phase-change material with a high dielectric constant, low losses, and strong tunability in the visible range [37, 38]. The permittivity dispersion of Sb_2_S_3_ (see Supplementary Information) indicates the broadband transparency of this material above the wavelength of 600 nm (frequency below 500 THz) in the amorphous phase.

We assume that the permittivity of the semiconductor core obeys the Drude–Lorentz model [28]:

(1) $$\varepsilon_{c}\;\left(\omega \right)=\varepsilon_{\infty }-\frac{f\omega_{0}^{2}}{\omega_{0}^{2}-\omega^{2}-i\Gamma \omega },$$

where *ε*_∞_ = 13.5 is the dispersionless part of the permittivity, *ω* is the angular frequency of light, *ω*_0_/2*π* = 474 THz is the resonant frequency of dipole transitions, Γ = 10^13^ s^−1^ is the polarization decay rate, and *f* is the oscillator strength related to the material gain *g* and speed of light *c* as [39] $f=gc\Gamma \sqrt{\varepsilon_{\infty }}/\omega_{0}^{2}$ . Chosen parameters are achievable by QDs and perovskites at room temperature [28]. The permittivity of 13.5 is typical for high-index semiconductors like GaAs, GaP, InAs, and others. The resonant frequency *ω*_0_ is optimized to achieve maximum spectral overlap with the laser mode.

This model allows us to study the core–shell nanostructure in optically passive (*g* ≤ 0) and active (*g* > 0) regimes. To this end, we chose the core radius of *R*_c_ = 109 nm and the radius of the shell of *R*_s_ = 125 nm. The optical response of the spherically symmetric core–shell nanostructure can be described by the generalized Mie theory [40]. The scattering cross-section (SCS) spectrum of the nanostructure can be calculated from the Mie multipole expansion [40, 41]:

(2) $$\text{SCS}=\frac{2}{{\left(kR_{\text{s}}\right)}^{2}}\overset{\infty }{\underset{l=1}{\sum }}\left(2l+1\right)\left(|a_{l}{|}^{2}+|b_{l}{|}^{2}\right),$$

where *l* is the multipole order, *k* = *ω*/*c* is the vacuum wavenumber, *a*_*l*_ and *b*_*l*_ are the frequency-dependent electric and magnetic multipole scattering coefficients.

According to the scattering theory, the optical properties of a system are defined by the eigenmodes of the system, which appear as poles of the scattering matrix coefficients at the corresponding complex eigenfrequencies [42]. Thus, to study the eigenmodes, eigenfrequencies, and their dynamics in the frequency domain, we calculate the SCS of the core–shell nanostructure in the complex frequency plane (*ω* = *ω*′ + *iω*″).

**Figure 2:**
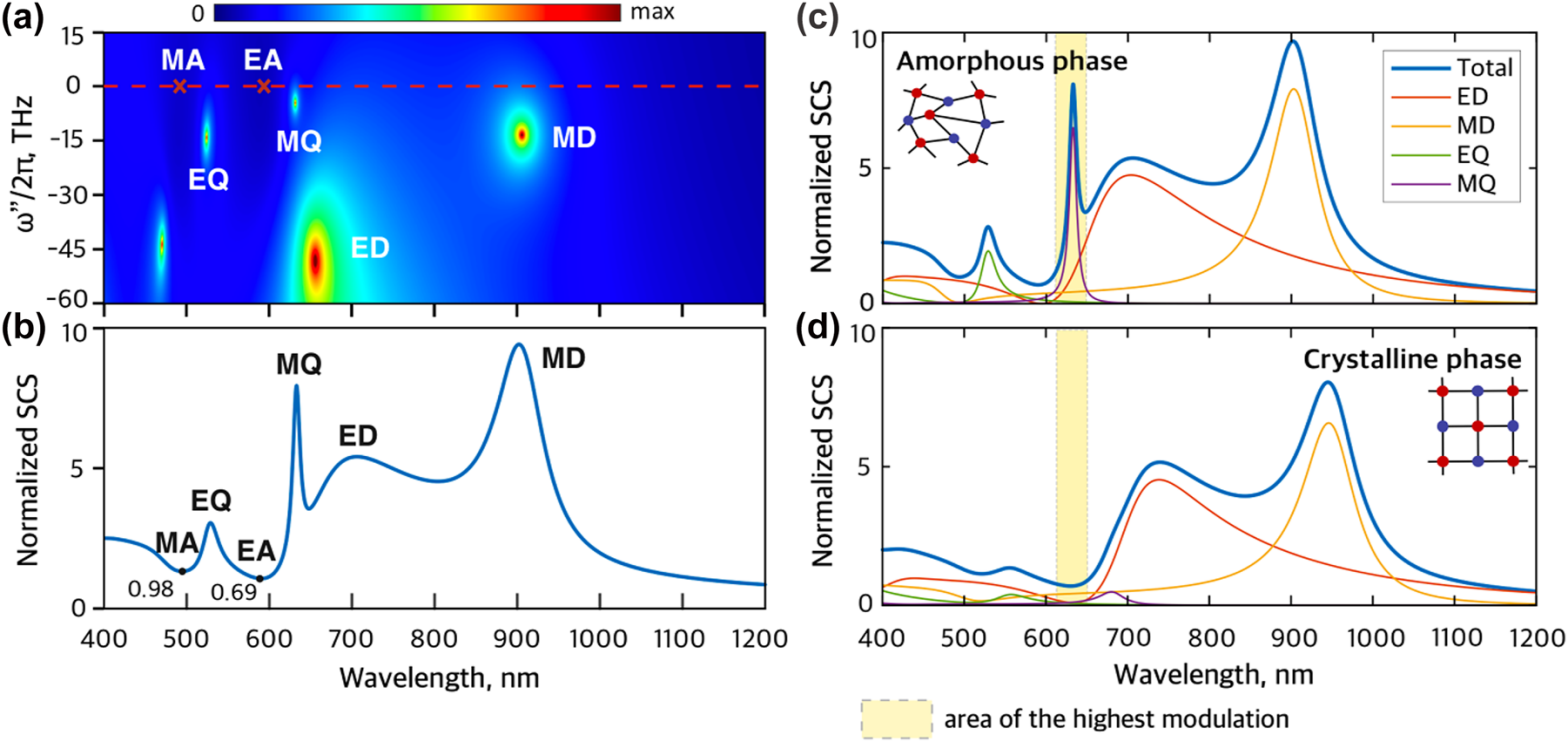
Passive core–shell nanostructure. (a) Normalized scattering cross-section (SCS) of a passive core–shell NP based on Sb_2_S_3_ shell and semiconductor core depending on wavelength (*λ* = 2*πc*/*ω*′) and imaginary part of frequency. The bright points (MD, ED, MQ, EQ) on the map represent poles of SCS, i.e., eigenfrequencies of the NP. The red marks (EA, MA) correspond to anapoles of the NP. (b) Normalized SCS at zero imaginary frequency depending on wavelength. (c, d) Multipole decomposition of normalized SCS of the core–shell NP in the amorphous (c) and crystalline (d) phases. The yellow area emphasizes the spectral range of the highest modulation of SCS due to the phase change.

The SCS of a passive (*g* = 0) core–shell nanostructure in an amorphous phase depending on the imaginary frequency *ω*″/2*π* and wavelength *λ* = 2*πc*/*ω*′ is shown in Figure 2(a). Due to passivity, the poles associated with the electric and magnetic multipoles of different order are observed in the lower complex frequency plane. The first four modes are magnetic dipole (MD) with an extracted eigenfrequency *ω*_MD_/2*π* = 330.6–13.25*i* THz, electric dipole (ED) with *ω*_ED_/2*π* = 456–47.75*i* THz, magnetic quadrupole (MQ) with *ω*_MQ_/2*π* = 474.0–4.5*i* THz, and electric quadrupole (EQ) with *ω*_EQ_/2*π* = 570–13.9*i* THz. The laser threshold can be estimated via the quality factor (Q-factor) of the mode of interest, or in other words, by the amount of gain required to deliver the pole to the real axis [39, 42]. Our calculations reveal that the MQ mode has the highest Q-factor of *Q* = 53 among the first four, making this mode a promising candidate for the nanolaser.

The red marks in Figure 2(a) designate the minima of the SCS located at the real axis (*ω*″ = 0). In literature, these minima are called non-scattering anapoles responsible for optical invisibility or cloaking [34, 35, 43]. Notably, the electric anapole (EA) is located near the MQ resonance, facilitating the tunability between MQ and EA states. The poles in the complex frequency plane project into resonances at the real axis, Figure 2(b). Also, note that anapole states are not eigenmodes of the system and, as such, they cannot lase, unlike poles [35]. We identify the multipole content of the investigated modes and anapoles of the core–shell in the amorphous and crystalline phases by the multipole expansion of the scattering spectra, Figure 2(c and d). The transformation of the Sb_2_S_3_ shell from amorphous to crystalline state leads to a switch of the scattering response around 633 nm wavelength (yellow area) from MQ resonance to non-scattering EA state.

**Figure 3:**
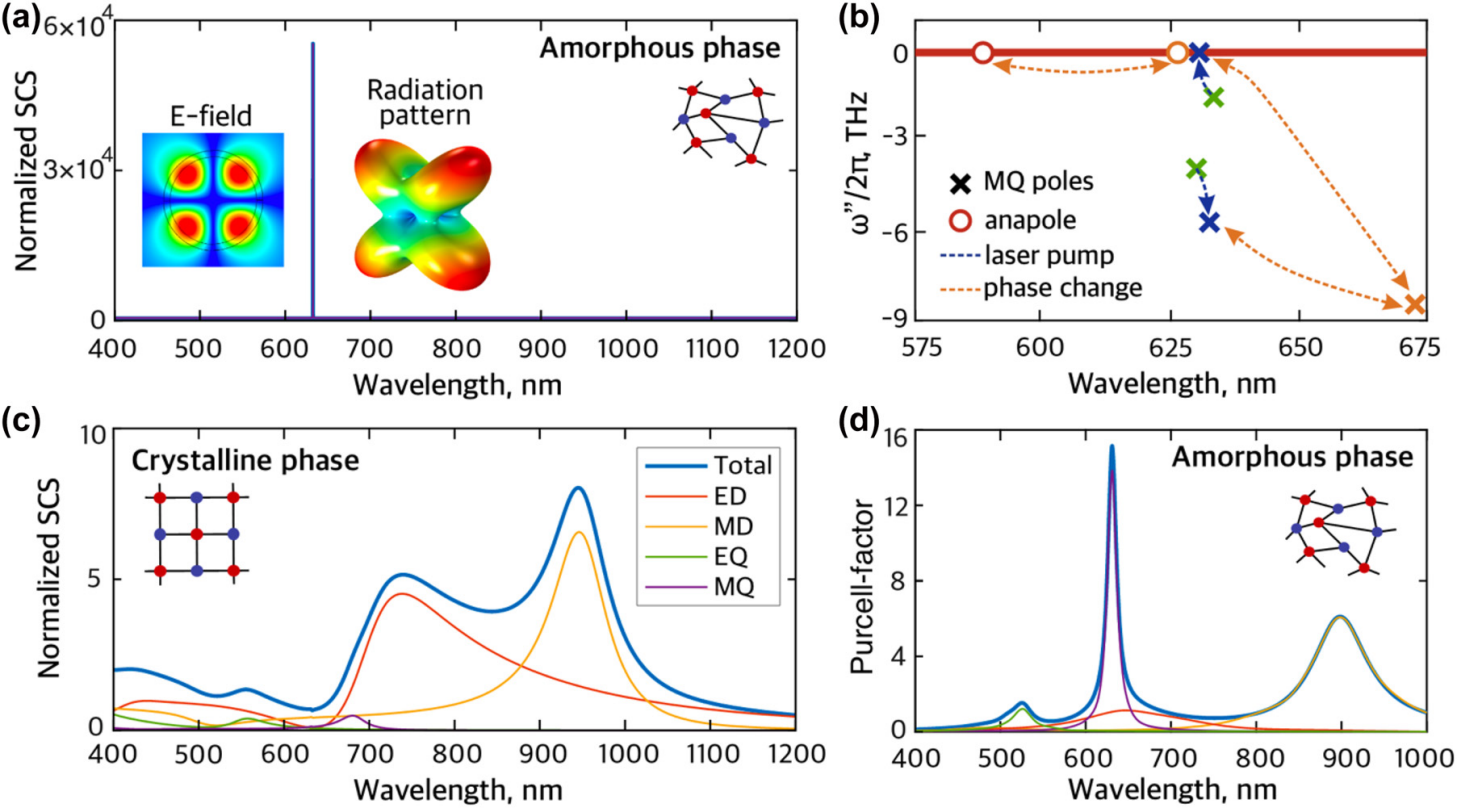
Active core–shell nanostructure. (a) Spectral dependence of normalized SCS of the NP with material gain *g* = 9.5 × 10^3^ cm^−1^ in the amorphous phase of the Sb_2_S_3_ shell and its multipole decomposition. The ultra-high peak of SCS at 633 nm in the amorphous phase corresponds to the laser regime. Insert shows the electric field profile and radiation pattern of the lasing mode (MQ). (b) Scheme of dynamics of poles and anapole (scattering zero) of the NP upon the laser pump and phase change. Different colors label poles and anapole corresponding to different states. Green corresponds to the system with the core in the amorphous state and ultimately small *g* → 0. Blue denotes the transition of the NP to the nanolaser (the phase remains amorphous) with *g* = 9.5 × 10^3^ cm^−1^ as the laser pump rises. Orange indicates reversible tuning between amorphous and crystalline phases. (c) Normalized SCS spectrum of the NP with *g* = 9.5 × 10^3^ cm^−1^ in the crystalline phase and its multipole decomposition. The pronounced dip in the crystalline phase is observed around 633 nm. (d) Purcell factor and its partial contributions from different modes of the NP with amorphous shell depending on wavelength.

Next, we study the transition to lasing in the core–shell nanostructure achieved by introducing the material gain (*g* > 0). The nanostructure is designed to maximize the overlap between the lasing MQ mode and material gain. The electric field distribution and the radiation pattern of this mode are shown in the insert of Figure 3(a). After introducing a non-zero value of the material gain, the NP modes start interacting with the material dipole transition, and the initial MQ mode splits into two modes. These modes appear as new distinct poles in the complex frequency plane. Upon increasing the material gain, the poles of the nanostructure shift from their initial positions marked by the green crosses in Figure 3(b). One reaches the real axis when the material gain reaches as high as *g*th = 9.5 × 10^3^ cm^−1^, marking the transition to lasing [28, 42]. The transition is accompanied by a sharpening of the SCS at the wavelength of laser radiation, as shown in Figure 3(a). Remarkably, pumping of the gain medium does not significantly change the scattering properties of the nanostructure in the crystalline phase that remains cloaked (Figure 3(c)).

The dynamics of the nanolaser is complicated since the polarization decay rate Γ of the dipole transition in the core is lower than the photonic decay rate of MQ mode *κ* = 5.65 × 10^13^ s^−1^. As a result, the dipole moment of the nanolaser gain medium cannot be adiabatically eliminated, which is sometimes referred to as a “superradiant regime” [44, 45]. For simulation of the input–output characteristic, we employ an approach that incorporates collective polarization correlations of emitters in the core into the densities of states and frequency-dependent population functions, thereby obtaining the spontaneous and stimulated emission rates in the convenient form of Fermi’s “golden rule” integrals [46]. Thus, the spontaneous (*R*_sp_) and stimulated (*R*_stim_) emission rates into the laser mode are evaluated as (see more details on the nanolaser model and coherence in Supplementary Information):

(3) $$R_{\text{sp}}=\frac{1}{4}G\Gamma \frac{n_{2}\left(1-n_{1}\right)}{n_{2}-n_{1}}\frac{\kappa +\Gamma -G/2}{{\left(\omega_{0}-\omega_{\text{MQ}}\right)}^{2}+{\left(\frac{\kappa +\Gamma -G/2}{2}\right)}^{2}}$$

(4) $$R_{\text{stim}}=\kappa N_{p}-R_{\text{sp}}$$

where *n*_2_ and *n*_1_ are occupation numbers of the excited and ground states of dipolar transition, $G=\kappa \frac{g}{g_{\text{th}}}\left[1+4{\left(\frac{\omega_{0}-\omega_{\text{MQ}}}{\kappa +\Gamma }\right)}^{2}\right]$ and the number of photons in the cavity is:

(5) $$N_{\text{p}}=\frac{\Gamma }{\Gamma +\kappa }\frac{n_{2}\left(1-n_{1}\right)}{n_{2}-n_{1}}\frac{g}{g_{\text{th}}-g}$$

Equations (3)–(5) imply that, in the steady state, the pole cannot cross or even reach the real axis since, as *g* approaches *g*th, the number of photons in the cavity grows infinitely. Also, from Eq. (3), it is evident that the beta-factor, i.e., the ratio of spontaneous emission into the laser mode to the total spontaneous emission into all modes, explicitly depends on the material gain, and therefore, it is not a parameter of the nanolaser.

To evaluate spontaneous emission into non-lasing modes, we have numerically determined the spectrum of Purcell enhancement *F*(*ω*) for a monochromatic emitter in the core and performed its multipole decomposition. The result, shown in Figure 3(d), is averaged over dipole orientations and positions within the core [47, 48]. From the Purcell enhancement spectrum, we were able to rigorously compute the beta-factor at transparency (*g* = 0) using: $\beta_{0}=\frac{\int_{0}^{\infty }F_{\text{MQ}}\left(\omega \right){\varepsilon^{\prime \prime }}_{c}\left(\omega \right)d\omega }{\int_{0}^{\infty }F\left(\omega \right){\varepsilon^{\prime \prime }}_{c}\left(\omega \right)d\omega }$ , where *F*_MQ_(*ω*) is the contribution of the magnetic quadrupole mode to *F*(*ω*) while $\varepsilon_{c}^{\prime \prime }\left(\omega \right)$ is the imaginary part of the dielectric function $\varepsilon_{c}^{\prime \prime }\left(\omega \right)$ , given by Eq. (1). After calculations, we obtain *β*_0_ = 0.841. The emission rate into the nonlasing MD, ED, and EQ modes is: $R_{\text{sp}}^{\text{nl}}=\frac{1-\beta_{0}}{4\beta_{0}}G\Gamma \frac{n_{2}\left(1-n_{1}\right)}{n_{2}-n_{1}}\frac{\kappa +\Gamma }{{\left(\omega_{0}-\omega_{\text{MQ}}\right)}^{2}+{\left(\kappa +\Gamma \right)}^{2}/4}$ . Finally, we include the nonradiative decay of excited emitters with the total rate *R*_nr_ = *Nn*_2_(1 − *n*_1_)/*τ*_nr_, where *N* = 1300 is the number of emitters and *τ*_nr_ = 1 ns is the nonradiative lifetime.

**Figure 4:**
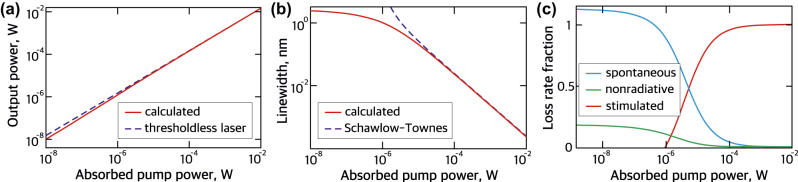
The nanolaser characteristics. (a) Light input–light output curve of the nanolaser. The blue dashed line corresponds to the characteristic of an ideal thresholdless nanolaser (for comparison). (b) Calculated linewidth of the nanolaser as a function of the pump power. (c) Fractions of spontaneous, stimulated emission rates and nonradiative decay in the total pump rate.

The input-output characteristic of the nanolaser is shown in Figure 4(a). The log–log plot does not exhibit a distinct kink which makes it similar to thresholdless lasers [49, 50]. This can be attributed to the high beta-factor close to 1, and strong Purcell enhancement in the strongly confined Mie resonance mode. The latter makes nonradiative recombination rates relatively small compared to the rates of radiative transitions. In a thresholdless nanolaser, it is impossible to recognize the transition to lasing based only on the input–output curve [51]. To make sure that we work with the correct part of the input–output curve, we have determined the emission linewidth as a function of the absorbed pump power, depicted in Figure 4(b), which clearly shows the onset of coherence at approximately 1 μW of absorbed pump power. On the plateau below 1 μW, the linewidth of about 2 nm is determined by the polarization dephasing rate of the gain medium rather than the lifetime of the cavity photons. Upon increasing the absorbed pump power above 1 μW, the laser linewidth decreases inversely proportional to the output power, which agrees with the Schawlow–Townes law [52] and experimentally observed linewidth dependence in thresholdless nanolasers [49, 50]. As indicated by Figure 4(c), the line narrowing coincides with the onset of stimulated emission in our structure, hence the input–output curve in Figure 4(a) shows the nanolaser operating in the spontaneous emission regime at low powers, the lasing regime dominated by the stimulated emission at high powers and transitional amplified spontaneous emission regime between them. At the same time, linewidth narrowing does not guarantee that the statistics of emitted photons would be Poissonian, as it should be for the coherent state [53]. The number of photons in the cavity [51] is estimated as $N_{\text{p}}\approx \sqrt{Ng_{th}/\left(dg/dn_{2}\right)}\approx 23$ , which corresponds to the absorbed pump power of 0.2 mW. However, more accurate calculation (see Supplementary Information) reveals that the laser crosses the coherence threshold at 50 μW, when the mean photon number in the laser mode is 5.7.

**Figure 5:**
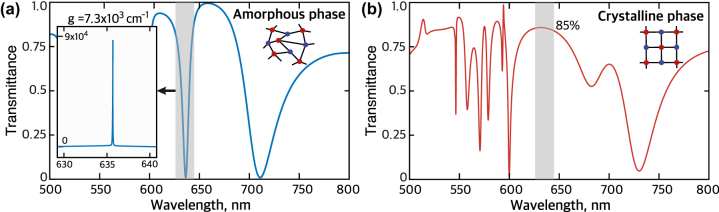
Cloaking a metasurface. (a) Transmittance spectrum of a metasurface composed of a square array of core–shell NPs in amorphous phase without gain. The insert shows the transmittance spectrum in the amorphous phase with gain of 7.3 × 10^3^ cm^−1^ in the shaded spectral region. (b) Transmittance spectrum of the metasurface in the crystalline phase with the same gain.

Finally, we arrange the core–shell NPs in a square lattice to thoroughly investigate the previously mentioned effects within a more practical metasurface-based transmitter application. The metasurface features a 600 nm period, with NP core and shell radii measuring 109 nm and 125 nm, respectively. We conduct comprehensive numerical analysis of the metasurface’s transmittance spectra in both amorphous and crystalline phases, incorporating and excluding gain, using COMSOL Multiphysics (see Figure 5). In the amorphous phase, the passive metasurface transmittance spectrum exhibits a distinct resonance near the magnetic quadrupole (MQ) resonance (Figure 5(a), shaded area). The resonance position is slightly shifted to the longer wavelengths due to interparticle interactions. The Q-factor of the resonance is increased with respect to the single NP MQ resonance, which is explained by the collective coupling of the NPs. This heightened Q-factor facilitates a lower gain lasing threshold.

The insert in Figure 5(a) shows the transmittance spectrum of the amorphous phase metasurface with gain of 7.3 × 10^3^ cm^−1^, around 25 % less than the initial gain of the single NP. The transmittance reveals an ultrasharp peak related to the nanolaser regime when the pole of the metasurface scattering matrix reaches the axis of real frequencies. However, after the phase change from amorphous to crystalline, the peak turns to a broad nonresonant response characterized by approximately 85 % transmittance from 625 nm to 645 nm wavelength, Figure 5(b). This allows us to conclude that the metasurface based on core–shell NPs can be reversibly switched from the emitting nanolaser regime to the transparent cloaking state.

While our study does not specify a particular semiconductor core material for the sake of generalization, the dielectric permittivity we used closely resembles that of indium phosphide (InP), and our design can be readily adapted to other active semiconductor materials, such as III–V semiconductors (GaAs with InGaAs quantum dots) or perovskite materials (CH_3_NH_3_PbI_3_, CsPbX_3_) [28].

## 3 Conclusions

In this work, we have put forth a nanolaser design that utilizes a semiconductor nanoparticle with gain, coated by a film of phase change material. This design enables the nanolaser to switch between lasing and cloaking modes at the same operating frequency. The cloaking phase of the nanostructure is linked to the anapole state. We have thoroughly examined the operational characteristics of the nanolaser. This nanolaser demonstrates thresholdless laser properties, lacking a noticeable kink in the input-light output curve due to the elevated betta and Purcell factors. Remarkably, the nanolaser functions in the superradiant regime because of the significant cavity losses. In this regime, the macroscopic dipole moment of the gain medium permits the attainment of the threshold at a reduced gain. A unique aspect of this regime is its narrower linewidth, determined by the emitters’ dephasing rate rather than the cavity quality factor. Furthermore, we have developed a reconfigurable metasurface composed of lasing-cloaking metaatoms, which can switch from laser radiation to a non-scattering state reversibly. Our findings hold potential for various photonic and nano-optical systems, particularly in situations where the light source needs the ability to transition to a transparent state.

## Supplementary Material

Supplementary Material Details
