# Supplementary material for: Switchable dual-mode nanolaser: mastering emission and invisibility through phase transition materials
Source: Nanophotonics. 2023 Sep 1;12(19):3729–36. doi: 10.1515/nanoph-2023-0249 (PMC11636156; doi:10.1515/nanoph-2023-0249)
Supplement: Supplementary file 1 — Supplementary Material Details [file j_nanoph-2023-0249_suppl_001.docx]

**Supplementary information**

**Switchable Dual-Mode Nanolaser: Mastering Emission and Invisibility through Phase Transition Materials**

Sergey Lepeshov^1^, Andrey Vyshnevyy^2^, and Alex Krasnok^3,4,*^

*^1^Department of Electrical and Photonics Engineering, DTU Electro, Technical University of Denmark, Building 343, DK-2800 Kgs. Lyngby, Denmark*

*^2^Emerging Technologies Research Center, XPANCEO, Dubai Investment Park 1, Dubai, United Arab Emirates*

*^3^Department of Electrical and Computer Engineering, Florida International University, Miami, FL 33174, USA*

*^4^Knight Foundation School of Computing and Information Sciences, Florida International University, Miami, FL 33199, USA*

**E-mail: akrasnok@fiu.edu*

**Optical properties of Sb_2_S_3_**

Sb_2_S_3_ is a prospective phase-change material with a high dielectric constant, low loss, and strong tunability in the visible range [1,2]. The permittivity dispersion of Sb_2_S_3_ is shown in Figure S1, and it indicates the broadband transparency of this material above the wavelength of 600 nm in the amorphous phase.


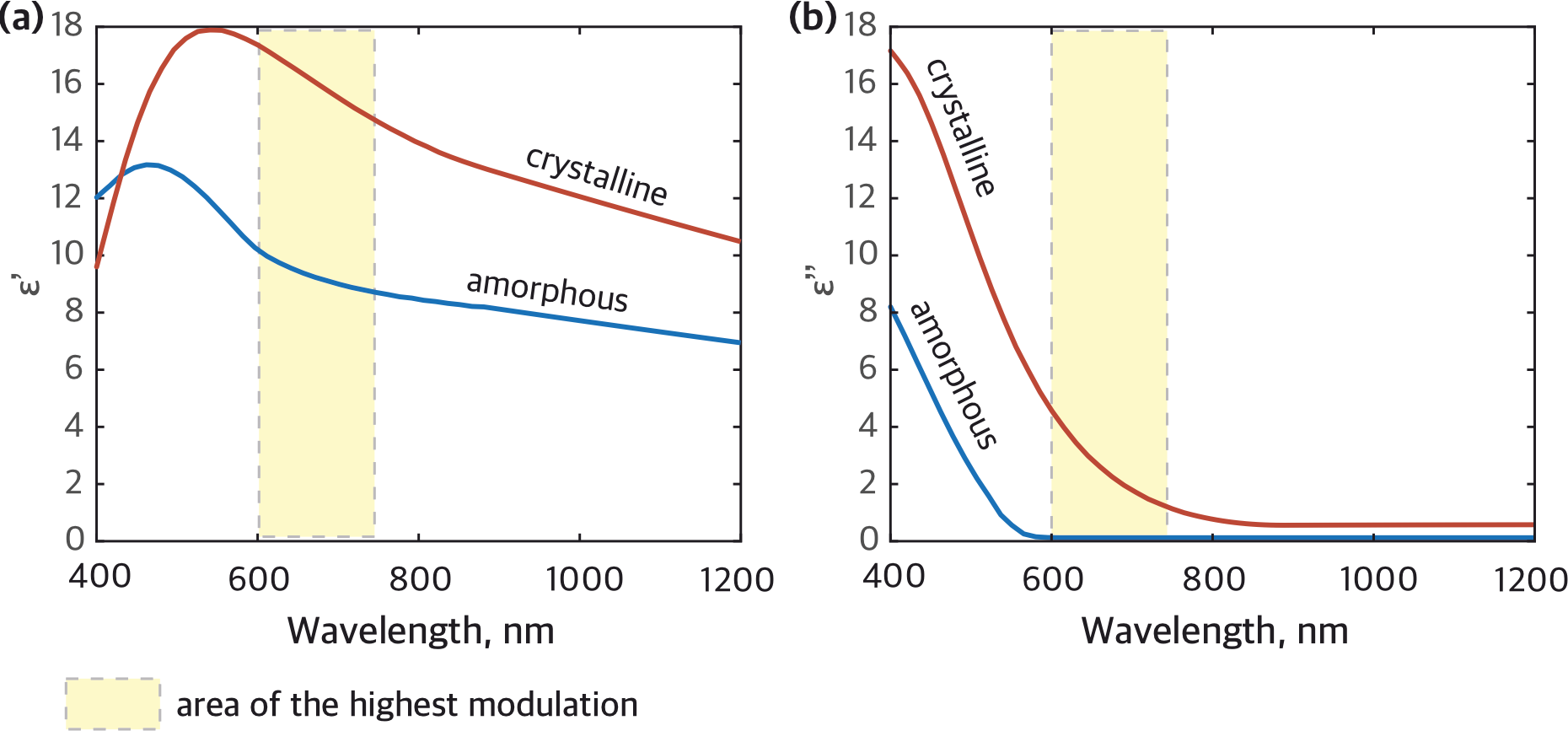


**Figure S1. –** **(a)** Real and **(b)** imaginary dielectric permittivity of Sb_2_S_3_ in amorphous (blue) and crystalline (red) phases.

**Nanolaser model**

To find the steady-state charachteristics of the nanolaser we employ a model based on the Keldysh technique for non-equilibrium Green’s functions (NEGF) and the Kadanoff–Baym equations [3]. For convenience, here we brief the main results of the paper and apply them to our nanolaser. From the Kadanoff–Baym equations we derive the spontaneous and stimulated transition rates in the form similar to Fermi’s golden rule integrals:

$R_{\mathrm{sp}}\approx\frac{2\pi N\left| g \right|^{2}}{\hbar^{2}}n_{2}\left( 1-n_{1} \right)\int d\omega\rho_{e}\left( \omega\right)\rho\left( \omega\right),$ (S1)

$R_{\mathrm{stim}}\approx\frac{2\pi N\left| g \right|^{2}}{\hbar^{2}}\left( n_{2}-n_{1} \right)\int d\omega\rho_{e}\left( \omega\right)S\left( \omega\right).$ (S2)

where *N* is the number of emitters, *n*_1_ and *n*_2_ are the populations of the ground and the excited states of emitters, *g* is the light-matter coupling constant, $\rho_{e}\left( \omega\right)=\frac{\Gamma/(2\pi)}{\left( \omega-\omega_{0} \right)^{2}+\left( \Gamma/2 \right)^{2}}$is the joint density of states of the quantum emitters, $\rho\left( \omega\right)$ is the optical density of states and $S\left( \omega\right)$ is the lineshape function which is related to the population of the laser mode as $\int S\left( \omega\right)d\omega=N_{p}$.

Equations (S1) and (S2) are fully equivalent to Fermi’s golden rule if we set $\rho\left( \omega\right)=\frac{\kappa/(2\pi)}{\left( \omega-\omega_{MQ} \right)^{2}+\left( \kappa/2 \right)^{2}}$ and $S\left( \omega\right)=N_{p}\rho\left( \omega\right)$, where $\omega_{MQ}$ and $\kappa$ are the angular frequency and the photon loss rate of the laser (magnetic quadrupole) mode. However, within NEGF framework

$\rho\left( \omega\right)=-\frac{1}{\pi}\mathrm{Im}\left[ G^{R}\left( \omega\right) \right]=-\frac{1}{\pi}\mathrm{Im}\left[ \frac{1}{\omega-\omega_{MQ}+i\kappa/2+\Pi_{\mathrm{int}}^{R}(\omega)} \right],$ (S3)

where

$\Pi_{\mathrm{int}}^{R}\left( \omega\right)=-\frac{\left| g \right|^{2}N\left( n_{2}-n_{1} \right)}{\omega-\omega_{0}+i\Gamma/2},$ (S4)

is the contribution of the light-matter interaction with the laser mode to polarization operator. The optical density of states coincides with the common Lorentzian function at transparency (*n*_2_=*n*_1_) but drastically differs from it when the gain medium is driven close to the lasing threshold. As a result the Purcell enhancement becomes gain-dependent. This effect is significant for “bad cavity” lasers where $\kappa$ is comparable with $\Gamma$.

The balance of photon generation and loss holds not only for the transition rates $R_{\mathrm{sp}}+R_{\mathrm{stim}}=\kappa N_{p}$ but also for their spectral densities

$\frac{2\pi N\left| g \right|^{2}}{\hbar^{2}}\left( n_{2}-n_{1} \right)\rho_{e}\left( \omega\right)\left[ \frac{n_{2}\left( 1-n_{1} \right)}{n_{2}-n_{1}}\rho\left( \omega\right)+S\left( \omega\right) \right]=\kappa S\left( \omega\right).$ (S5)

which determines the lineshape function. Set of equations (S1-5) fully determines the steady state of the laser at a given population inversion. Also, they describe effects of frequency pulling, superradiance and yield the threshold population inversion consistent with the semisclassical Maxwell–Bloch model.

Insering the functions $\rho\left( \omega\right)$ determined from Eqs. (S3,4) into Eq. (S1) we find the total spontaneous emission rate

$R_{\mathrm{sp}}=\frac{1}{4}G\Gamma\frac{n_{2}\left( 1-n_{1} \right)}{n_{2}-n_{1}}\frac{\kappa+\Gamma-G/2}{\left( \omega_{0}-\omega_{\mathrm{MQ}} \right)^{2}+\left( \frac{\kappa+\Gamma-G/2}{2} \right)^{2}},$ (S6)

where $G=\kappa\frac{g}{g_{\mathrm{th}}}\left[ 1+4\left( \frac{\omega_{0}-\omega_{\mathrm{MQ}}}{\kappa+\Gamma} \right)^{2} \right]$ with $g_{\mathrm{th}}$ is the threshold material gain. The stimulated ^emission rate is determined as $R_{\mathrm{stim}}=\kappa N_{p}-R_{\mathrm{sp}}$, where

$N_{p}=\int S\left( \omega\right)d\omega=\frac{\Gamma}{\Gamma+\kappa}\frac{n_{2}\left( 1-n_{1} \right)}{n_{2}-n_{1}}\frac{g}{g_{\mathrm{th}}-g}$. (S7)

**Nanolaser coherence**

To determine the coherence threshold, we have employed Langevin equation approach. In particular, we have estimated the threshold conditions using the approximate equation (13) of work [4] that can be reformulated in terms of threshold photonic population as $N_{p}\approx\sqrt{Ng_{th}/(dg/dn{}_{2})}\approx23$. However, it is worthy to employ a more accurate equation (9) which reads

$g^{(2)}\left( 0 \right)=1+\frac{A^{2}D_{\mathrm{rr}}+2ACD_{\mathrm{sr}}+\left( AD+BC+C^{2} \right)D_{\mathrm{ss}}}{\left( B+C \right)\left( AD+BC \right)N_{p}^{2}}$ (S8)

where *A*, *B*, *C*, and *D* are the coefficients of rate equations linearized close to the stationary point and *D*_ij_ are the diffusion coefficients that characterize strengths of the Langevin forces.


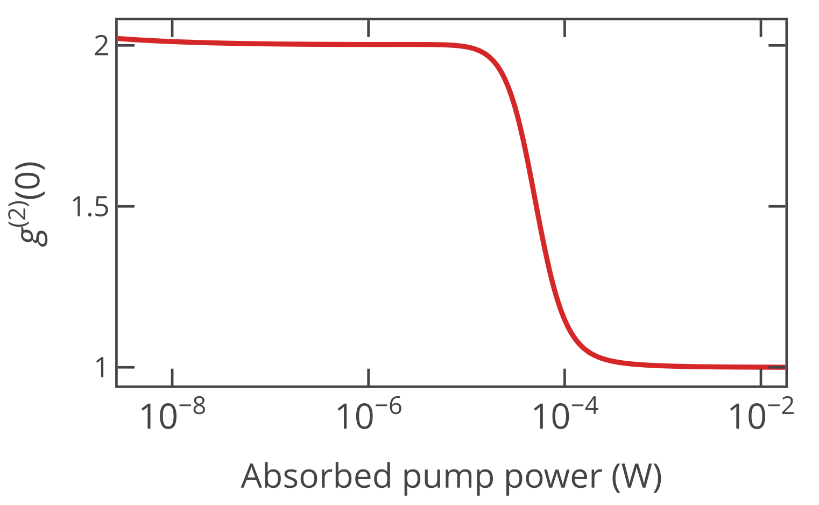


**Figure S2. –** Second-order coherence function calculated using Eq. (S8). At threshold $g^{(2)}\left( 0 \right)=1$.5 the absorbed pump power reaches about 50 μW which corresponds to 5.6 photons inside the mode.

**References:**

[1] S. K. Chamoli, G. Verma, S. C. Singh, and C. Guo, *Phase Change Material-Based Nano-Cavity as an Efficient Optical Modulator*, Nanotechnology **32**, (2021).

[2] W. Dong, H. Liu, J. K. Behera, L. Lu, R. J. H. Ng, K. V. Sreekanth, X. Zhou, J. K. W. Yang, and R. E. Simpson, *Wide Bandgap Phase Change Material Tuned Visible Photonics*, Adv. Funct. Mater. **29**, 1806181 (2019).

[3] A. A. Vyshnevyy, *Gain-Dependent Purcell Enhancement, Breakdown of Einstein’s Relations, and Superradiance in Nanolasers*, Phys. Rev. B **105**, 085116 (2022).

[4] A. A. Vyshnevyy and D. Y. Fedyanin, *Lasing Threshold of Thresholdless and Non-Thresholdless Metal-Semiconductor Nanolasers*, Opt. Express **26**, 33473 (2018).
